# Weak selection for resistance to quorum sensing inhibition during multiple host infection cycles

**DOI:** 10.1093/ismejo/wrae251

**Published:** 2024-12-17

**Authors:** Qian Yang, Tom Defoirdt

**Affiliations:** Department of Biotechnology, Center for Microbial Ecology and Technology (CMET), Ghent University, Frieda Saeysstraat 1, 9052 Gent, Belgium; Department of Biotechnology, Center for Microbial Ecology and Technology (CMET), Ghent University, Frieda Saeysstraat 1, 9052 Gent, Belgium

**Keywords:** quorum sensing, quorum quenching, antivirulence therapy, fitness

## Abstract

Quorum sensing (QS) inhibition is a promising novel approach to control bacterial infections. However, it is not clear whether QS inhibition will impose selective pressure for the spread of resistance against QS inhibition in pathogen populations. Previous research tried to answer this question by using synthetic growth media, and this revealed that whether or not resistance will spread completely depends on the environment in which it is studied. Therefore, the spread of resistance should be studied in the environment where it ultimately matters: in vivo during infection of a host. Here, using QS inhibitor-susceptible and -resistant mimics, we show that resistance to QS inhibition does not spread in host-associated populations of *Vibrio campbellii* during up to 35 cycles of infection and transmission if the initial frequency of the resistance is low in the pathogen population, whereas it further increases to 100% if it is already prevalent. However, even in the latter case, the resistance spreads at a slower pace than resistance to antibiotics spreads under the same conditions.

The modes of action of currently used antibiotics are primarily variations on a single theme: killing or inhibiting growth [1]. Such modes of action impose strong selective pressure for resistance development. Indeed, resistant mutants are able to grow in the presence of antibiotics, whereas wild type cells are either killed or inhibited in their growth. As a result, resistance to antibiotics is spreading rapidly, and bacteria showing clinically relevant resistance to antibiotics consistently appear within as little as a few years after first use [1]. Because of this, the development of novel effective and sustainable strategies to control bacterial diseases is one of the major societal challenges of this moment. One of the strategies that is intensively investigated, is the inhibition of quorum sensing (QS), bacterial cell-to-cell communication with small signal molecules. Indeed, laboratory experiments documented that QS inhibition is an effective strategy to control diseases caused by various bacterial pathogens of plants, animals and humans [2–5], although clinical or field experiments are still needed to further confirm this.

In contrast to antibiotics, QS inhibition has long been believed to exert minimal selective pressure, thus rendering the spread of resistance unlikely (e.g. [6–8]). However, this assumption was too optimistic because it was based on experiments performed in nutrient-rich synthetic growth media (where QS is not essential) [9]. Indeed, *Pseudomonas aeruginosa* was later shown to evolve resistance to halogenated furanones, the most intensively studied QS inhibitors, in an environment where QS confers a strong selective advantage [10]. Specifically, QS inhibitor-resistant mutants were obtained in a synthetic growth medium containing adenosine as the sole carbon source, and growth on adenosine is dependent on the QS-regulated intracellular nucleoside hydrolase enzyme in *P. aeruginosa*. It has been argued that the spread of resistance will depend on whether QS affects fitness predominantly via private goods (i.e. products that only benefit the producer cell, such as nucleoside hydrolase in *P. aeruginosa*) or via public goods (i.e. products that can benefit other cells in addition to the producer cell, e.g. extracellular protease that determines growth on extracellular protein as sole carbon source in *P. aeruginosa*) [11]. Indeed, in an environment in which fitness depends on QS-regulated public goods, such as a synthetic growth medium with extracellular protein as the sole carbon source, resistance to QS inhibition does not spread [11].

From the above, it is clear that whether or not resistance to QS inhibition will spread in synthetic growth media depends on the specific composition of these media. The key question that remains however, is whether resistance will spread in the clinic, and this question cannot be answered by performing experiments using synthetic growth media. A better approach would be to investigate the spread of resistance in a host during multiple cycles of infection and transmission [12–14]. However, thus far, this has not yet been studied for any bacterial pathogen.

In this study, we aimed at investigating the spread of resistance to QS inhibition in a host, using the QS model pathogen *Vibrio campbellii. V. campbellii* contains a three-channel QS system, which has been shown to control bioluminescence and different virulence-related phenotypes, including flagellar motility [15] and the extracellular Vhp metalloprotease [16]. Furthermore, an active three-channel QS system is required for full virulence of the bacterium to different hosts, including brine shrimp (*Artemia franciscana*) [17]. Central in the three-channel QS system of *V. campbellii* is the LuxO protein [18], and the point mutation LuxO D47E renders the QS system completely nonresponsive to the QS signal molecules [19]. Therefore, this strain is dark and we used this mutant to mimic the QS output of wild type *V. campbellii* treated with a 100% effective and 100% specific QS inhibitor. This strain is further denoted as “sensitive genotype”. Wild type *V. campbellii*, on the other hand, is luminescent and was used to mimic the QS output of a mutant that is completely resistant to such an inhibitor when treated with the inhibitor. This strain is further denoted as “resistant genotype”.

We first aimed to determine the spread of resistance in synthetic growth media in order to confirm that also in *V. campbellii*, the environment determines whether resistance will spread or not. In order to determine the spread of resistance to QS inhibition in these media, we used two initial ratios of resistant genotype:sensitive genotype (1:100 and 1:1), simulating a scenario in which resistance just arose and therefore is still rare, and a scenario in which resistance is already prevalent, respectively. We determined the frequency of the resistant genotype in the colony center, colony edge and whole colony, respectively, both on soft LB35 agar, where QS-regulated private goods (i.e. flagellar motility – Fig. S1 [15]) were expected to have a dominant impact on fitness, and on soft M9-casein agar, where QS-regulated public goods (i.e. extracellular protease [16]) were expected to have a dominant impact on fitness. We found that resistance indeed spreads on LB35 soft agar, since the frequency of the resistant genotype increased in the colony edges and whole colonies, both in case of low and high initial frequencies (Fig. 1A and B and Fig. S2). On the other hand, resistance is negatively selected on M9-casein soft agar, since the frequency of the resistant genotype decreased in the colony edges and whole colonies (Fig. 1C and D and Fig. S2). Therefore, whether or not resistance to QS inhibition in *V. campbellii* spreads is indeed dependent on the environment and therefore, infection experiments are needed in order to obtain a relevant indication of whether resistance will spread in real life.

**Figure 1:**
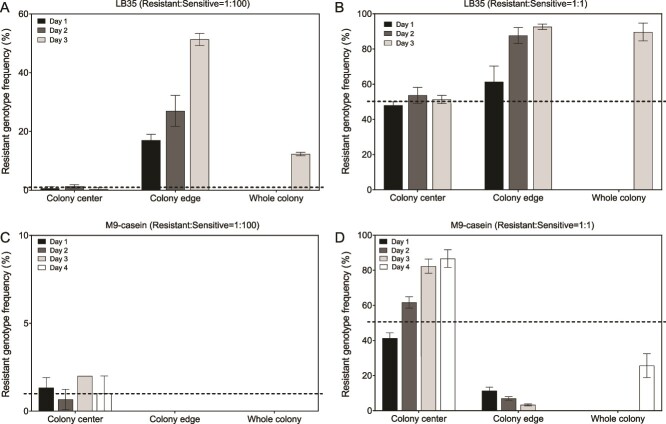
**Selection of resistance to quorum sensing inhibition in synthetic environments.** Selection of resistance in *V. campbellii* populations growing on LB35 soft agar (panels A and B) and M9-casein soft agar (panels C and D), both when resistance is initially scarce (resistant genotype: sensitive genotype 1:100; panels a and C) and when resistance is already prevalent in the beginning (resistant genotype: sensitive genotype 1:1; panels B and D). The graphs show the frequency of the resistant genotype in the *V. campbellii* population in the colony centers, colony edges and whole colonies. The dotted lines indicate the initial resistant genotype frequencies. Error bars indicate the standard deviations of three biological replicates.

We subsequently monitored the spread of the resistant genotype in *V. campbellii* populations during multiple cycles of infection of brine shrimp larvae (see Supplemental Material for a detailed description of the procedure), both for shrimp-associated vibrios and vibrios in the external environment. Brine shrimp were challenged by adding mixed *V. campbellii* populations to the rearing water. Similar to the in vitro experiments, we used two initial ratios of resistant genotype:sensitive genotype (1:100 and 1:1). At the end of each cycle of infection (48 h post-challenge), a 10% (v/v) fraction of the rearing water was used to inoculate the subsequent brine shrimp cultures to initiate the next cycle. In case the resistant genotype was present at a low initial frequency, its frequency in the host slightly increased in cycles 8–11 and 6–8 in trial 1 and 2, respectively, but did not further increase after that, and even decreased again in trial 2 (Fig. 2). The same evolution was observed for the vibrios in the rearing water (Fig. S3). These results indicate that in a scenario in which a new mutation arises that renders the pathogen resistant to QS inhibition, the resistant mutant would not spread in the population when treated with a QS inhibitor.

**Figure 2:**
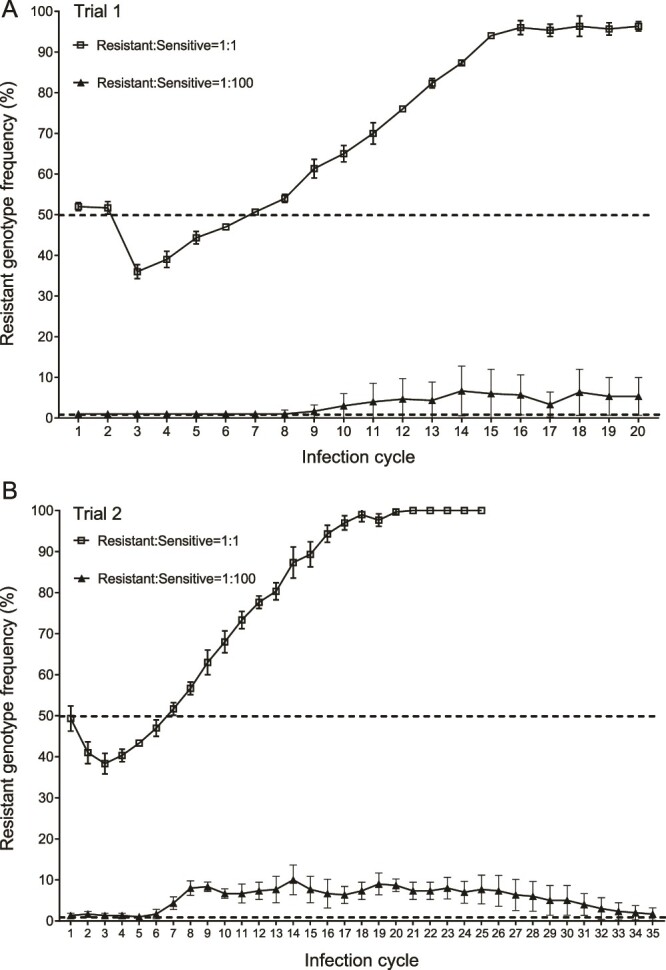
**Selection of resistance to quorum sensing inhibition in host-associated *Vibrio campbellii* populations during multiple cycles of infection of gnotobiotic brine shrimp.** Selection of resistance was monitored in host-associated *V. campbellii* populations both when it was initially scarce (resistant genotype: sensitive genotype 1:100) and when it was already prevalent in the beginning (resistant genotype: sensitive genotype 1:1). The graphs show the frequency of the resistant genotype in the host-associated *V. campbellii* populations during 20 cycles (trial 1; panel A) and 35 cycles (trial 2; panel B) of infection, respectively. The dotted lines indicate the initial resistant genotype frequencies. Error bars represent the standard deviations of three brine shrimp cultures.

In case the resistant genotype was present at a relatively high initial frequency, in contrast, its frequency further increased and reached 100% in the host after 16 and 18 cycles of infection in trial 1 and 2, respectively (Fig. 2). The same evolution was observed for vibrios in the rearing water (Fig. S3). This indicates that if resistance to QS inhibition is prevalent, then it will increase further until it reaches 100% when the population is treated with a QS inhibitor. Such a scenario could occur, for instance, in case QS inhibition would be used in attempts to control infection caused by a pathogen in which overexpression of a multidrug efflux pump has been selected by prior antibiotic treatment and as a result of that is prevalent in the population. Indeed, it was previously reported that overexpression of a multidrug efflux pump can cause cross-resistance to a QS inhibitor in *P. aeruginosa* [10]. The fact that resistance to QS inhibition increases rather than decreases in the host environment suggests that in a host environment (or during transmission), QS-regulated private goods rather than QS-regulated public goods determine fitness.

Even in the scenario where the resistant genotype was prevalent from the start, resistance to QS inhibition spread more slowly than antibiotic resistance. Indeed, we performed an additional experiment in which we monitored the frequency of kanamycin resistance in host-associated *V. campbellii* populations under kanamycin treatment and found that it reached 100% after two cycles of infection already (Fig. S4), even if the initial frequency was low (1%).

It needs to be acknowledged that the experimental set-up using resistant and sensitive mimics reflects an idealized scenario in which QS inhibition is 100% effective and 100% specific. Real QS inhibitors, in contrast, will likely not be 100% effective and 100% selective [12], and this can have an impact on the selection of resistance. Nevertheless, our work indicates that the strategy of QS inhibition itself does result in a weaker selective pressure in infected hosts than the use of antibiotics. However, in case a QS inhibitor would be used that affects growth in some manner in addition to inhibiting QS, then the selective pressure for resistance development will be higher and resistance will likely evolve faster than what we observed here. In this regard, it has recently been demonstrated that *P. aeruginosa* quickly evolves resistance to QS-inhibiting furanones in biofilms grown in synthetic sputum medium [20]. It would be very interesting to find out whether this would also occur during consecutive cycles of infection of a host. In the future, QS inhibitors that show higher specificity than brominated furanones (which are highly reactive molecules) will likely be identified. The more specific such inhibitors will be, the more similar their selective pressure for resistance will be to what we observed in this study.

## Supplementary Material

Supplemental_material_wrae251

## Data Availability

Data supporting the findings of this work are available within the paper and its Supplementary Material file.

## Materials & Correspondence

Correspondence and material requests should be addressed to Tom Defoirdt.
