# Supplementary material for: Weak selection for resistance to quorum sensing inhibition during multiple host infection cycles
Source: ISME J. 2024 Dec 17;18(1):wrae251. doi: 10.1093/ismejo/wrae251 (PMC11684082; doi:10.1093/ismejo/wrae251)
Supplement: Supplemental_material_wrae251 [file supplemental_material_wrae251.pdf]

Weak selection for resistance to quorum sensing inhibition during multiple host infection cycles – Supplemental material

Qian Yang and Tom Defoirdt

Materials and Methods

Bacterial strains, media, and growth conditions

In this study, two *Vibrio campbellii* strains derived from wild type strain BB120 (=ATCC BAA-1116) were used. Strain JAF136 (LuxO WT linked to Kan^R [1]) was used as quorum sensing inhibitor resistant mimic (further denoted resistant genotype), i.e. the quorum sensing system of this strain is fully functional, simulating the activity of the quorum sensing system of a mutant that would be completely resistant to a quorum sensing inhibitor in the presence of this inhibitor. This strain is luminescent. Strain JAF548 (LuxO D47E linked to Kan^R [1]) was used as quorum sensing inhibitor susceptible mimic (further denoted sensitive genotype), i.e. this strain still produces the quorum sensing signal molecules, but is nonresponsive to them, thus simulating the activity of the quorum sensing system of wild type bacteria that are fully sensitive to the activity of a quorum sensing inhibitor in the presence of this inhibitor. This strain is dark.

The strains were grown in Luria Broth with 35 g/L Instant Ocean sea salt (LB35) or M9 salts medium supplemented with sodium chloride to 20 g/L and sodium caseinate as the sole carbon source at a concentration of 5 g/L (M9-casein). Strains were inoculated from frozen stocks (stored in glycerol at -80°C) into LB35 broth and acclimated by passaging for 24 h in M9-casein prior to the initiation of experiments in M9-casein. Liquid cultures were incubated at 28 °C under constant agitation (120 rpm). Cell densities were measured spectrophotometrically at 600 nm. Viable cell counts were determined by plate counting on LB35 agar plates.

In vitro competition on soft agar plates

Motility assays were conducted on soft agar plates containing 3 g/L agar. Prior to inoculation onto the soft agar plates, strains were grown overnight in LB35 broth and then transferred and subsequently grown in fresh LB35 or M9-casein medium for 12h or 24h, respectively, to ensure acclimation. The strains were mixed in different ratios of resistant genotype:susceptible genotype (1:1 and 1:100) at a total density of 10^7 cells/ml. Next, 3 µl aliquots of the mixed cultures were carefully spotted at the center of soft agar plates and the plates were subsequently incubated upright at 28°C. Samples were collected daily throughout the experiment, with center samples picked from the initial inoculation

point on the motility plate, and edge samples obtained from the visible boundary of the expanding colonies. On the third day (LB35) and fourth day (M9-casein), respectively, the entire colonies were excised from the plates, suspended in 10 ml of LB35 or M9-casein liquid medium, thoroughly vortexed until homogenous, and subsequently diluted and plated on LB35 agar. The frequency of the resistant genotype was determined by calculating the ratio between the number of luminescent colonies and the total number of colonies.

***In vivo* competition in brine shrimp cultures**

Gnotobiotic brine shrimp experiments were performed as described previously [2] with some modifications. Specifically, after 28 h post-hatching, batches of 30 sterile brine shrimp larvae at developmental stage instar II (with functional digestive tracts) were counted and transferred to sterile 50-ml tubes containing 30 ml of filtered and autoclaved artificial seawater (containing 35 g/l of Instant Ocean sea salt). A suspension of autoclaved *Aeromonas* sp. LVS3 bacteria was added as feed at the start of the challenge test at 10^7 cells/ml. To initiate the first cycle of infection, the resistant and susceptible genotype strains were mixed in different ratios (1:1 and 1:100) and were added to the brine shrimp rearing water at a total density of 10^6 CFU/ml. Each treatment was carried out in six replicates. After 24h of incubation, samples of both the rearing water and 30 live brine shrimp larvae were harvested from half of the cultures (i.e. three replicate cultures from each treatment), to determine the frequency of the resistant genotype in the water and in the shrimp. To determine the frequency of the resistant genotype in the shrimp-associated populations, brine shrimp larvae were carefully collected on a sterile 100-µm cell strainer and rinsed twice with 10 ml autoclaved and filtered artificial seawater. Subsequently, the larvae were transferred to Eppendorf tubes containing sterile glass beads and submerged in autoclaved artificial seawater to reach a final volume of 1 ml. Then, the larvae were homogenized by bead beating at 2000 rpm for 70 s. Supernatants were collected, serially diluted and plated on LB35 agar. Rearing water samples were also serially diluted and plated on LB35 agar to assess the frequency of the resistant genotype in the populations in the external environment. The frequency of the resistant genotype was determined by calculating the ratio between the number of luminescent colonies and the total number of colonies. At the end of each cycle of infection (48h post-challenge), a 10% (v/v) fraction of the rearing water was used to inoculate the subsequent brine shrimp cultures. This was repeated 20 times and 35 times for the first and second repetition of the experiment, respectively.

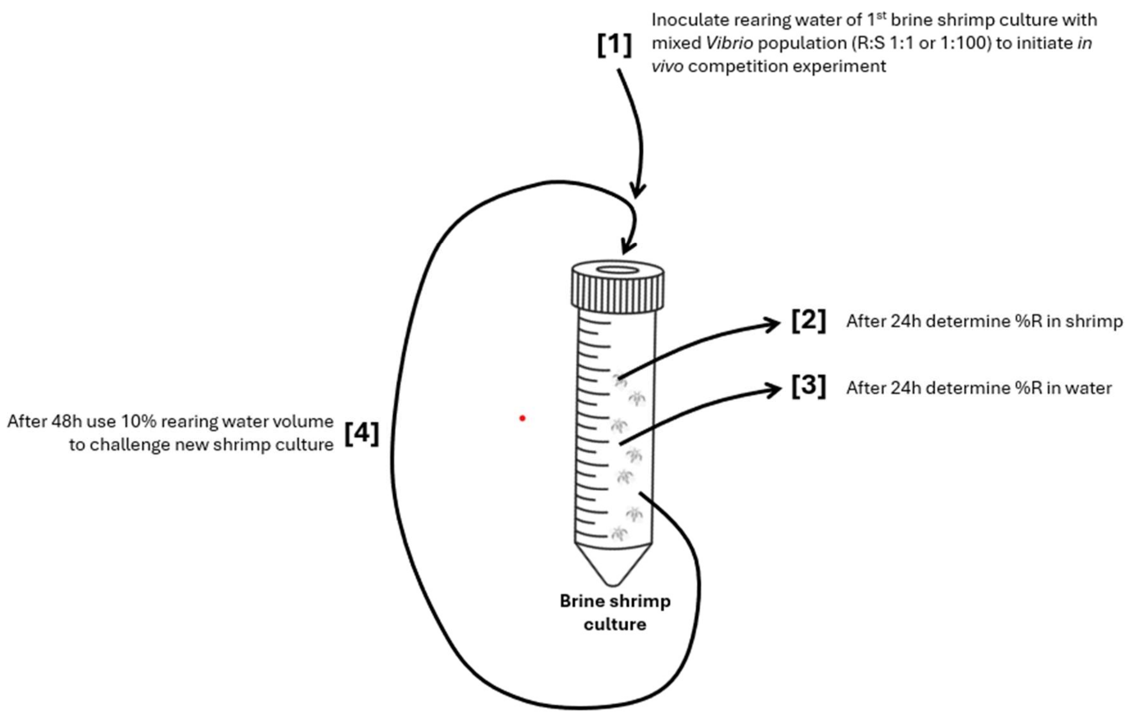

Figure: Schematic representation of the *in vivo* competition experiment. R: resistant; S: sensitive.

To compare the pace of the spread of resistance to quorum sensing inhibition with that of antibiotic resistance, another *in vivo* competition experiment was performed with the kanamycin-resistant strain JAF136 (containing LuxO WT linked to Kan^R [1]) and the kanamycin-susceptible wild type strain BB120. The kanamycin-resistant and kanamycin-susceptible strains were mixed in a ratio of 1:100. Kanamycin was added to the brine shrimp rearing water at a concentration of 300 µg/ml. Samples of both homogenized brine shrimp larvae and rearing water were serially diluted and plated on LB35 agar with and without 300 µg/ml of kanamycin, respectively. The frequency of the kanamycin-resistant strain was determined by calculating the ratio between the number of colonies growing on LB35 agar with kanamycin and the number of colonies growing on LB35 agar without kanamycin.

References

[1] Freeman, J.A. & Bassler, B.L. A genetic analysis of the function of LuxO, a two-component response regulator involved in quorum sensing in *Vibrio harveyi*. *Molecular Microbiology* **31**, 665-77 (1999).

[2] Defoirdt, T. *et al.* Quorum sensing-disrupting brominated furanones protect the gnotobiotic brine shrimp *Artemia franciscana* from pathogenic *Vibrio harveyi, Vibrio campbellii,* and *Vibrio parahaemolyticus* isolates. *Applied and Environmental Microbiology* **72**, 6419-23 (2006).

Supplementary figures

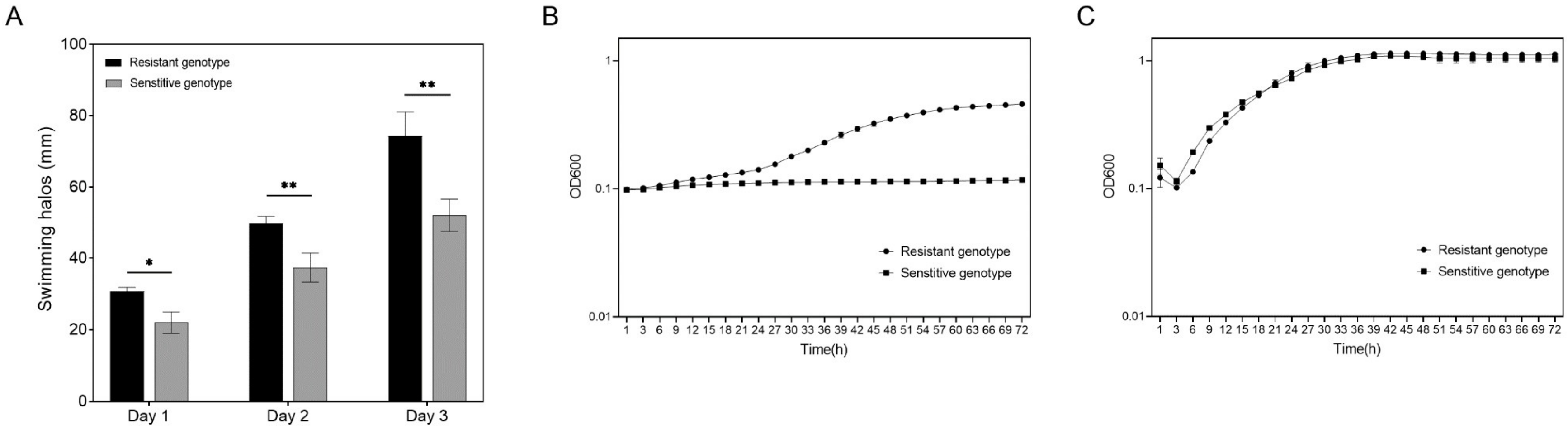

Figure S1. Quorum sensing controls the production of private and public goods in *Vibrio campbellii*, but has no impact on fitness in an environment where quorum sensing is not essential. (A) Motility zones of resistant genotype and sensitive genotype on soft LB35 agar. **(B)** Growth of resistant genotype and sensitive genotype in M9-Casein broth. **(C)** Growth of resistant genotype and sensitive genotype in LB35 broth.

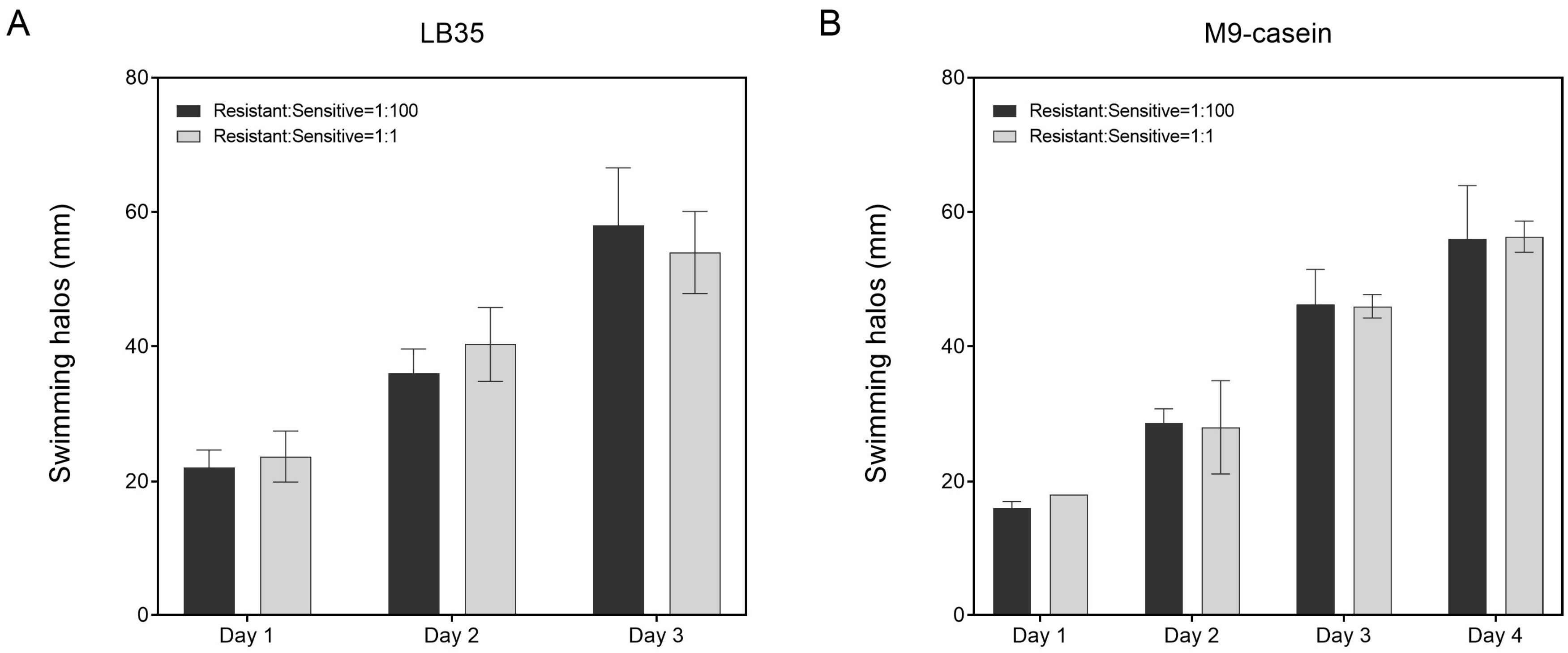

Figure S2. Motility halo dimaters of the mixed cultures on LB35 soft agar and M9-casein soft agar during the *in vitro* resistance evolution experiment.

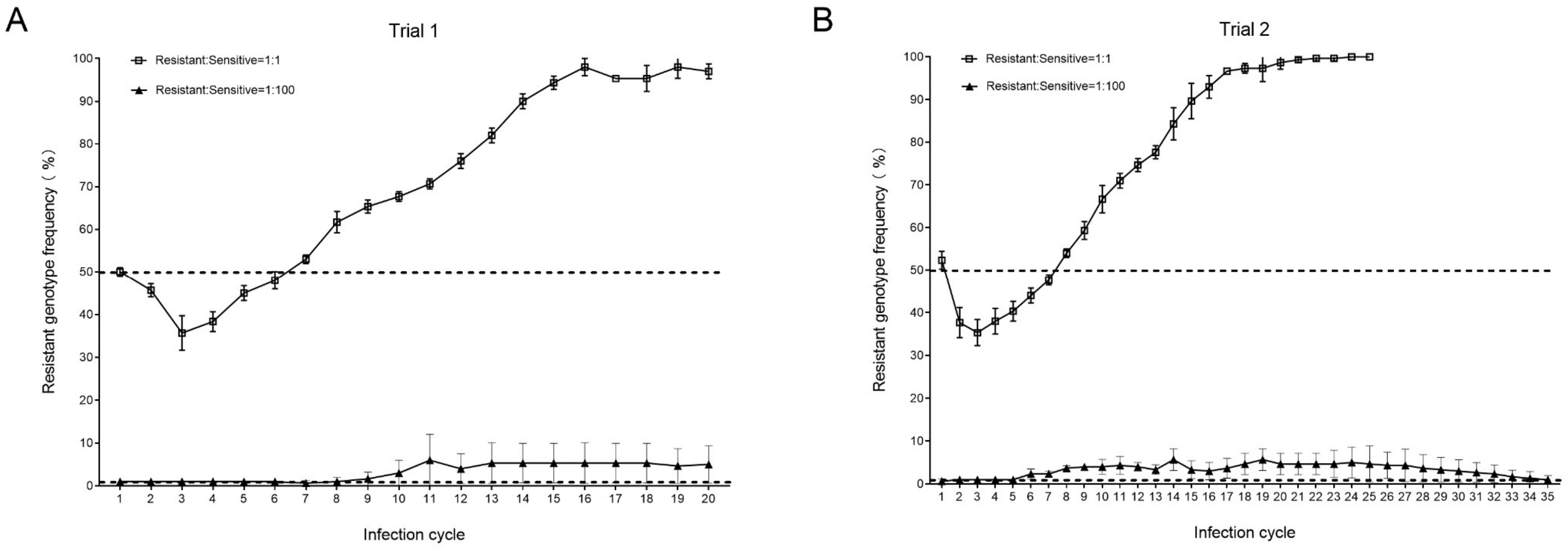

Figure S3. Selection of resistance to quorum sensing inhibition in *Vibrio campbellii* populations in the external environment. Selection of resistance was monitored in the brine shrimp rearing water, both when it was initially scarce (resistant:sensitive 1:100) and when it was already prevalent in the beginning (resistant:sensitive 1:1). The graphs show the frequency of the resistant genotype in the *V. campbellii* population in the brine shrimp rearing water during 20 cycles (Experiment 1; panel A) and 35 cycles (Experiment 2; panel B) of infection, respectively. The dotted lines indicate the initial resistant genotype frequencies. Error bars represent the standard deviation of three brine shrimp cultures.

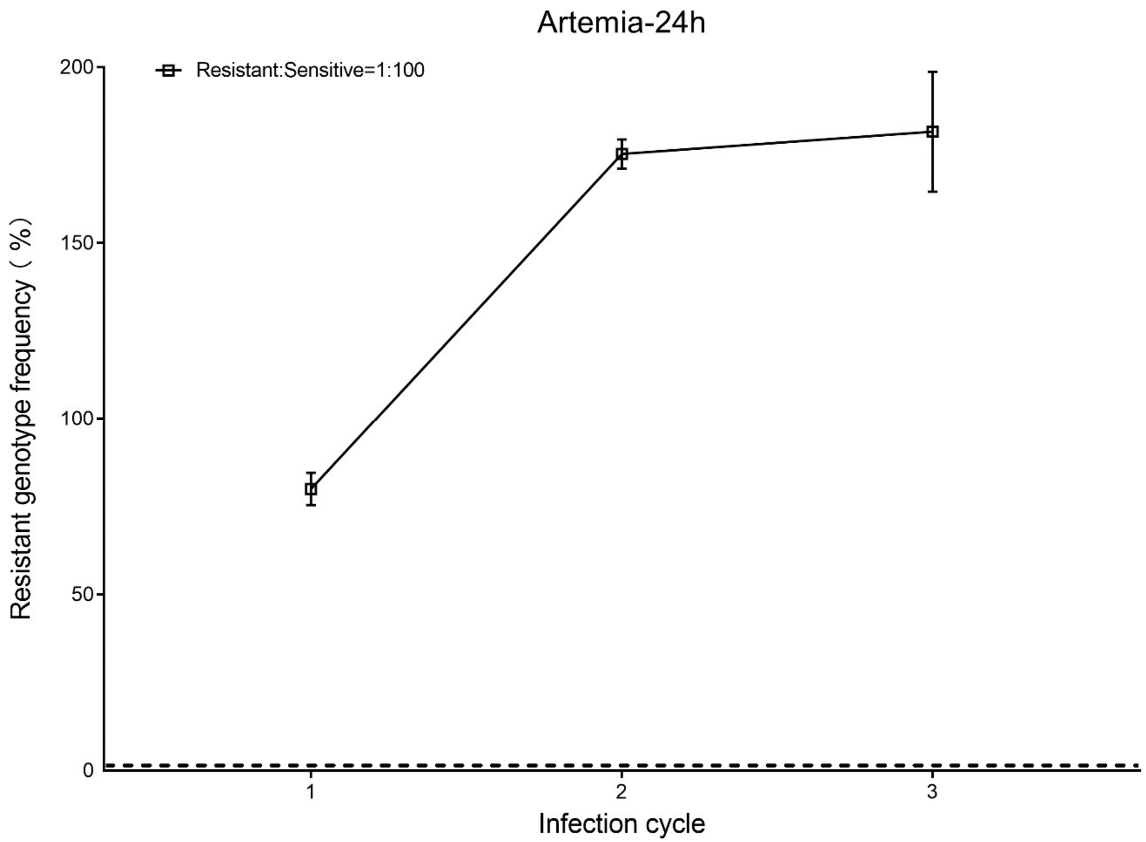

Figure S4. **Selection of resistance to the antibiotic kanamycin in host-associated *Vibrio campbellii* populations.** Selection of kanamycin resistance was monitored during multiple cycles of infection of gnotobiotic brine shrimp under kanamycin treatment, starting from an initial ratio resistant:sensitive of 1:100. The graph shows the frequency of the resistant genotype in the *V. campbellii* population that is associated with brine shrimp during 3 cycles of infection. The dotted line indicates the initial resistant genotype frequency. Error bars represent the standard deviation of three brine shrimp cultures.
